# Modeling the Solubility of Monoterpenoids with Hybrid and Predictive Thermodynamic Tools

**DOI:** 10.1021/acs.iecr.2c03991

**Published:** 2023-03-13

**Authors:** Sérgio M. Vilas-Boas, Isabella W. Cordova, Dinis O. Abranches, João A. P. Coutinho, Olga Ferreira, Simão P. Pinho

**Affiliations:** † Centro de Investigação de Montanha (CIMO), Instituto Politécnico de Bragança, Campus de Santa Apolónia, 5300-253 Bragança, Portugal; ‡ Laboratório para a Sustentabilidade e Tecnologia em Regiões de Montanha, Instituto Politécnico de Bragança, Campus de Santa Apolónia, 5300-253 Bragança, Portugal; § CICECO − Aveiro Institute of Materials, Department of Chemistry, University of Aveiro, 3810-193 Aveiro, Portugal

## Abstract

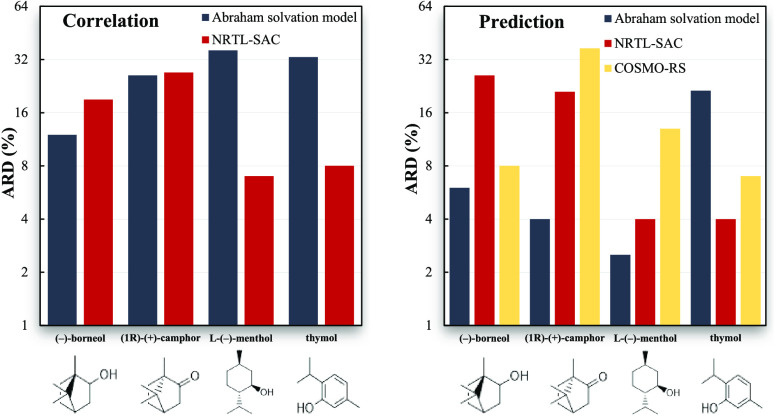

The Abraham and NRTL-SAC semipredictive models were employed to represent the solubility of (−)-borneol, (1R)-(+)-camphor, l-(−)-menthol, and thymol in water and organic solvents, using data measured in this work and collected from the literature. A reduced set of solubility data was used to estimate the model parameters of the solutes, and global average relative deviations (ARDs) of 27% for the Abraham model and 15% for the NRTL-SAC model were obtained. The predictive capability of these models was tested by estimating the solubilities in solvents not included in the correlation step. Global ARDs of 8% (Abraham model) and 14% (NRTL-SAC model) were obtained. Finally, the predictive COSMO-RS model was used to describe the solubility data in organic solvents, with ARD of 16%. These results show the overall better performance of NRTL-SAC in a hybrid correlation/prediction approach, while COSMO-RS can produce very satisfactory predictions even in the absence of any experimental data.

## 1 Introduction

Monoterpenoids constitute a structurally diverse class of compounds abundantly found in essential oils.^1,2^ Due to their often pleasant aromas^3^ and diverse biological and pharmacological activities,^2,4^ monoterpenoids have been increasingly exploited in the fragrance, cosmetic, food, and pharmaceutical industries.^1,5^ To design and optimize extraction and purification processes to obtain monoterpenoids from their natural matrices, the knowledge of equilibrium properties, such as solubility and partition coefficient data, in different solvent systems is extremely valuable. From the pool of relevant monoterpenoids, four commercially important representatives were selected: (−)-borneol, (1R)-(+)-camphor, l-(−)-menthol, and thymol.

Camphor is one of the chief representatives of bicyclic monoterpene ketones, having several applications in the pharmaceutical field.^6,7^ Present in *Cinnamomum camphora* species, camphor has been widely used in traditional medicine to treat several diseases, such as rheumatism, chest congestion, muscle pain, asthma, and bronchitis.^6^ In modern medicine, this substance is an ingredient of analgesics and rubefacients, although its overdose ingestion or skin penetration might cause severe toxic effects or even death.^7^ Another bicyclic monoterpenoid, borneol, is a monoterpene alcohol used as a fragrance ingredient in personal care and cleaning products.^8^ This compound also presents relevant pharmacological and biological properties, such as analgesic, antibacterial, anti-inflammatory, and antioxidant activities.^9^ It has been extensively investigated as a potential permeation enhancer to facilitate drug delivery through different biological barriers.^9−11^

Menthol is widely employed as an aroma ingredient in cosmetics, perfumes, household cleaners, and detergents.^12,13^ It is a cyclic monoterpene alcohol produced by numerous plants from the *Lamiaceae* family. It is broadly used in medicine to relieve localized pain and respiratory disorders due to its “cool” characteristic sensation triggered by its interactions with thermoreceptors in the human skin.^13,14^ Recently, the potential of menthol and thymol for the formulation of some innovative deep eutectic solvents^15−17^ has been demonstrated. Thymol, a phenolic monoterpenoid, presents various applications in medicine, dentistry, food, and agrochemical industries.^18^ Because of its numerous bioactivities (antioxidant, anti-inflammatory, antimicrobial, and antifungal), thymol has been extensively studied as a therapeutical agent for wound healing.^19−21^ Besides, thymol brings several nutritional and biological benefits when incorporated as a feed additive in fish production.^22^

Following our previous studies on water solubilities^23^ and octanol-water partition coefficients^24^ of a diverse set of monoterpenoids, this work aims to investigate the solubility of (−)-borneol, (1R)-(+)-camphor, l-(−)-menthol, and thymol in seven organic solvents (acetonitrile, 1-butanol, ethanol, ethyl acetate, hexane, R-(+)-limonene, and 1,2-propanediol). For (−)-borneol, (1R)-(+)-camphor, and thymol, solubility measurements were carried out at 298.2 and 313.2 K, while for l-(−)-menthol with a melting point of 315.6 K,^25^ experiments were only performed at 298.2 K. The solubility of (−)-borneol in water, not examined in our previous works, was also obtained here at 298.2 and 313.2 K.

Finally, three well-established thermodynamic models were applied to describe the solubility change with solvent and temperature. The Abraham solvation model^26,27^ was applied to calculate the monoterpenoids solubility in organic solvents at 298.2 K,^28−30^ while the Nonrandom Two-Liquid Segment Activity Coefficient (NRTL-SAC) model,^31,32^ and the Conductor-like Screening Model for Real Solvents (COSMO-RS)^33−35^ were employed to represent the available solubility data in water and organic solvents at different temperatures.^30,36,37^

## 2 Experimental Section

### 2.1 Chemicals

All of the monoterpenoids and organic solvents used in the solubility experiments are listed in Table 1, along with their chemical structure, CAS number, source, and purity. For the monoterpenoids, melting temperature (*T*_m_) and melting enthalpies (Δ*_m_H*) are also provided. The organic compounds were used as received, and the solids were kept in the desiccator to prevent water contamination. Ultrapure water (resistivity of 18.2 MΩ·cm, free particles ≥0.22 μm, and total organic carbon <5 μg·dm^–3^) was used in the solubility studies of (−)-borneol.

**Table 1:**
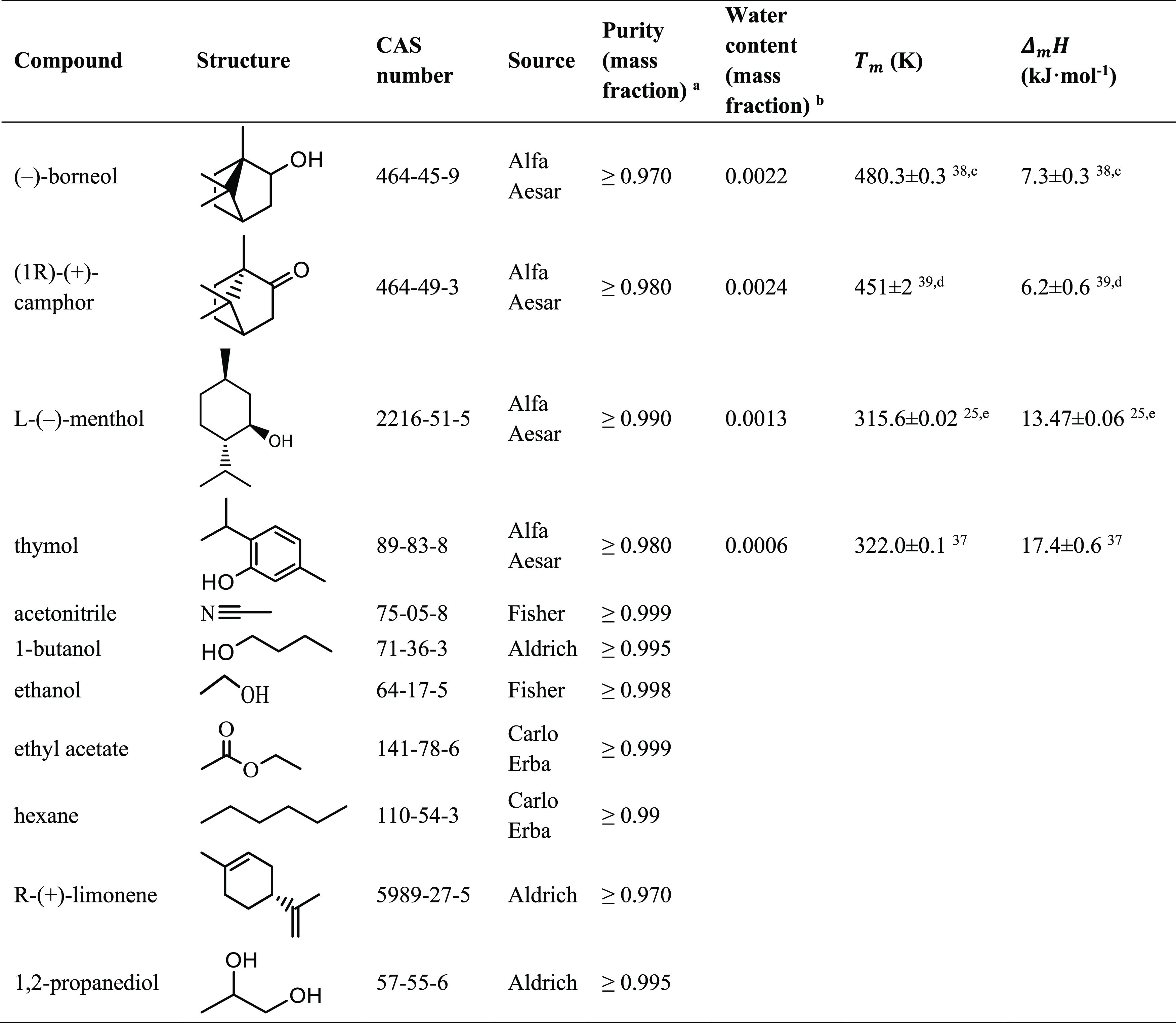
Chemical Structure, CAS Number, Source, Mass Purity, Melting Temperature (*T*_m_), and Melting Enthalpy (Δ_*m*_*H*) of the Solutes and Organic Solvents Studied in This Work

a Values correspond to the minimum mass fraction purities granted by the supplier.

b Water content (mass fraction) measured by Karl–Fisher titration (Metrohm, 831 KF Coulometer).

c (−)-Borneol presents a solid–solid transition (II → I) at *T*_II →__I_ = 347.9 ± 0.3 K; Δ*H*_II → I_ = 3.2 ± 0.3 kJ·mol^–1^. ^38^

d (+)-Camphor presents two low-temperature solid–solid transitions (III → II and II → I) at *T*_III → II_ = 244 ± 1 K; Δ*H*_III → II_ = 10 ± 3 kJ·mol^–1^ and *T*_II → I_ = 369 ± 6 K; Δ*H*_II → I_ = 0.19 ± 0.07 kJ·mol^–1^.^39^

e Melting properties correspond to the stable α phase of l-menthol.^25^

### 2.2 Solubility Experiments

The solubility experiments were conducted using the isothermal shake-flask method, which was described in detail previously.^40^ In brief, around 50 mL of saturated solutions of monoterpenoid and solvent, containing small amounts of solid in excess, were prepared in all glass flasks covered with aluminum foil to prevent light degradation. The flasks were placed on magnetic stirrers (Cimarec I micro stirrer, Thermo Fisher) inside a heated water circulating bath (TC120, Grant) equipped with a precise temperature control system (±0.1 K). Preliminary experiments demonstrated that the equilibrium was achieved after 24 h of continuous stirring, and 16 h of settling was enough for most suspended solid particles to deposit.

At least three samples, between 0.2 and 0.5 cm^3^, were collected using previously heated glass syringes coupled to nylon or polytetrafluoroethylene filters (0.45 μm pore diameter). After, the samples were analyzed by UV–vis spectroscopy or gas chromatography (GC). The analytical procedures are presented in Section S1 in the Supporting Information (SI). Each reported solubility value is the average of at least three independent readings.

## 3 Modeling

### 3.1 Abraham Solvation Model

The Abraham solvation model describes the ratio of the solubilities (in molar concentration basis) of a solute in an organic solvent (*S*_s_) and in water (*S*_w_) through the following linear free energy relationship (LFER)^27^
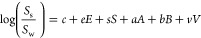
1where the uppercase descriptors (*E*, *S*, *A*, *B*, and *V*) and the lowercase descriptors (*c*, *e*, s, *a*, *b*, and *v*) represent the Abraham descriptors for the solute and solvent, respectively.

Abraham’s research group has already reported the model parameters for several solvents.^26,27,41,42^ Regarding the solute descriptors, *V*, the solute’s McGowan characteristic molecular volume, and *E*, the solute excess molar refractivity, can be directly obtained from the solute chemical structure and refractive index.^27,43^ The other parameters, *S* (dipolarity/polarizability), *A* (overall hydrogen-bond acidity), and *B* (overall hydrogen-bond basicity), are frequently regressed using a set of experimental solubility and/or partition coefficient data.

### 3.2 NRTL-SAC Model

The semipredictive NRTL-SAC model has been extensively applied to describe the solubility of biomolecules in water and organic solvents^40,44−50^ and was described in detail by Chen and co-authors.^31,32^ In this approach, the activity coefficient of solute *i* is calculated as the sum of the combinatorial (γ_*i*_^C^) and the residual (γ_*i*_^R^) contributions
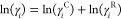
2

In summary, the model characterizes the solute and solvent molecules by four conceptual segments representing different surface interactions: hydrophilicity (*X*), hydrophobicity (*Z*), polar-attractive (*Y*^–^), and polar-repulsive (*Y*^+^). These parameters are available in the literature for many solvents,^31,32,45,50^ including those addressed in this work. Therefore, only the solute descriptors are required to estimate the solubilities with the NRTL-SAC, which will be regressed using a small set of the experimental solubility data.

The solubility of a solute (*i*) in a liquid mixture can be described by^51^
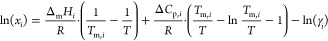
3where *x*_*i*_ is the mole fraction solubility of solute *i* at temperature *T*, Δ_m_*H*_*i*_ and *T*_m,*i*_ are its melting enthalpy and temperature, respectively, Δ*C*_p,*i*_ is its heat capacity change upon melting, γ_*i*_ is its activity coefficient, and *R* is the ideal gas constant. The contribution of the Δ*C*_p,*i*_ term on the calculation of *x*_*i*_ is often negligible,^51^ and eq 3 can be simplified to
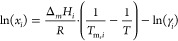
4

Equation 4 is frequently used to describe the solid–liquid equilibrium of a pure solid substance in a liquid mixture when no solid–solid transition between *T* and *T*_m,*i*_ occur. Whenever such transitions are present, the modified form of eq 4 is recommended^52,53^
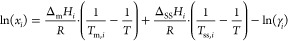
5where *T*_ss,*i*_ is the transition temperature and Δ_SS_*H*_*i*_ is its enthalpy.

In this work, eq 5 was applied to describe the solubilities of (−)-borneol and (1R)-(+)-camphor with the NRTL-SAC or COSMO-RS models due to the presence of solid–solid transitions between 298.2 K and melting.^38,39^ For l-(−)-menthol and thymol, eq 4 was employed. All of the phase transition properties of the solutes considered in the solubility estimations were collected from the open literature^25,37−39^ and are listed in Table 1.

### 3.3 COSMO-RS Model

COSMO-RS estimates activity coefficients in liquid mixtures, which can be easily coupled with eqs 4 and 5 to estimate solubilities, by computing pair-wise interaction energies between the molecular surface segments of all mixture components. The screened charges of these segments, which are encoded in the so-called sigma surfaces and profiles, are computed using DFT with the COSMO solvation model.

COSMO-RS was employed in this work through its implementation in the COSMOtherm software package (version 21.0),^35,54^ with the BP_TZVPD_FINE_21.ctd parametrization. This parametrization requires sigma surfaces optimized using the def2-TZVPD basis set, the BP-86 DFT functional, and the COSMO solvation model with infinite permittivity. When available, these were collected from the COSMOtherm TZVPD-FINE database. Those sigma profiles not available in the database (monoterpenoids and some solvents) were computed using the TmoleX (version 4.5) software package,^55^ coupled with COSMOconfX 2021 for initial conformer generation (BP-TZVPD-FINE-COSMO+GAS_18 template). All conformers obtained were considered to perform the calculations in COSMOtherm. More details are available in the COSMOtherm reference manual.^56^

### 3.4 Assessment of the Deviations

The deviations between the experimental and calculated solubilities were assessed by the average relative deviation (ARD)
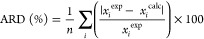
6where superscripts “exp” and “calc″ mean the experimental and calculated solubilities, respectively, *n* is the total number of data points, and *i* covers all of the solvent systems for a given solute.

## 4 Results and Discussion

### 4.1 Solubility Measurements

The solubilities of (−)-borneol, (1R)-(+)-camphor, l-(−)-menthol, and thymol in the selected organic solvents (acetonitrile, 1-butanol, ethanol, ethyl acetate, hexane, R-(+)-limonene, and 1,2-propanediol) are presented in Table 2 along with the ideal solubility values. The aqueous solubilities of the four monoterpenoids, and the experimental activity coefficients, calculated from the mole fraction solubilities listed in Table 2 using eq 5, are presented in Tables S1 and S2 in the SI, respectively. At this point, it is critical to observe the impact of solid–solid transitions on the ideal solubility and, therefore, on the activity coefficients. While for (1R)-(+)-camphor the impact is almost null, which is consistent with the very low transition enthalpy, for (−)-borneol, at 298.2 K, the ideal solubility would be 0.327, 20.2% higher than that presented in Table 2 amplifying, generally, the deviations from ideality.

**Table 2:** Experimental Solubilities (in Mole Fraction) of the Monoterpenoids in Organic Solvents at 298.2 and 313.2 K^a^,^b^

	(−)-borneol		(1R)-(+)-camphor		l-(−)-menthol	thymol	
solvent	298.2 K	313.2 K	298.2 K	313.2 K	298.2 K	298.2 K	313.2 K
ideal^c^	0.272	0.333	0.422	0.478	0.740	0.595	0.832
acetonitrile	0.0451 ± 0.0004	0.0781 ± 0.0002	0.5872 ± 0.0008	0.7212 ± 0.0002	0.4047 ± 0.0019	0.7059 ± 0.0017	0.9001 ± 0.0004
1-butanol	0.3121 ± 0.0010	0.3480 ± 0.0020	0.4541 ± 0.0020	0.5347 ± 0.0025	0.7103 ± 0.0029	0.6663 ± 0.0045	0.8298 ± 0.0018
ethanol	0.2977 ± 0.0007	0.3256 ± 0.0010	0.3795 ± 0.0006	0.5215 ± 0.0031	0.6510 ± 0.0022	0.6586 ± 0.0016	0.8435 ± 0.0018
ethyl acetate	0.2365 ± 0.0013	0.2956 ± 0.0008	0.5868 ± 0.0023	0.7035 ± 0.0037	0.6805 ± 0.0041	0.7090 ± 0.0052	0.8713 ± 0.0033
hexane	0.0887 ± 0.0012	0.1426 ± 0.0012	0.5237 ± 0.0016	0.5477 ± 0.0057	0.6927 ± 0.0056	0.3380 ± 0.0029	0.8203 ± 0.0044
R-(+)-limonene	0.1443 ± 0.0012	0.2263 ± 0.0024	0.6278 ± 0.0025	0.6790 ± 0.0016	0.6906 ± 0.0043	0.4797 ± 0.0034	0.8071 ± 0.0027
1,2-propanediol	0.1236 ± 0.0008	0.1568 ± 0.0014	0.0894 ± 0.0022	0.1163 ± 0.0003	0.6179 ± 0.0012	0.6959 ± 0.0029	0.8456 ± 0.0038

a Temperature and pressure standard uncertainties are *u*(*T*) = 0.10 K and *u*_r_(*p*) = 0.05, respectively.

b Standard deviations are placed after the ± sign.

c Calculated for borneol and camphor using the phase transition properties presented in Table 1.

The good consistency of the solubility values is demonstrated by the low coefficient of variation, lower than 2.5% for organic systems and 3.7% for the (−)-borneol/water system.

Excepting (1R)-(+)-camphor/water system, where the observed solubility at 313.2 K is 3 times lower than the value obtained at 298.2 K,^24^ in all other systems studied at both temperatures, the solubility increases with the temperature. In general, the solubilities of the monoterpenoids in organic solvents are much higher than in water, with differences varying from 2 orders of magnitudes for (−)-borneol to 4 orders of magnitude for l-(−)-menthol. The ideal mole fraction solubility values listed in Table 2 (higher than 0.27) are much closer to the solubilities in organic solvents, showing better affinity with the solutes. Indeed, due to the hydrophobic nature of monoterpenoids, the activity coefficients of all solutes in water were much higher than 1, ranging from 2.7 × 10^3^ for (−)-borneol at 298.2 K to 1.7 × 10^4^ of l-(−)-menthol at 298.2 K.

Concerning the monoterpene alcohols, l-(−)-menthol is more soluble in all of the organic solvents than (−)-borneol, at 298.2 K. This behavior is in line with the ideal solubility trend (*x*_ideal, menthol_ ≈ 2.7·*x*_ideal, borneol_). Moreover, they exhibit positive deviations from ideality for most studied binary mixtures, except for (−)-borneol in ethanol (at 298.2 K) or 1-butanol. Among the organic solvents, the highest and the lowest solubilities of both solutes were observed for 1-butanol and acetonitrile, respectively.

Excepting for the solubilities of (1R)-(+)-camphor in 1,2-propanediol and ethanol (at 298.2 K), the solubilities are higher than the ideal value, and consequently, the activity coefficients are lower than 1. The highest solubilities occur in R-(+)-limonene, ethyl acetate, and acetonitrile, whereas 1,2-propanediol is the organic solvent with the poorest affinity to this monoterpene ketone. Taking into account the Hansen solubility parameters,^57^ it can be said that solvents of intermediate polarity and small hydrogen-bond character favor camphor solubilization. Different from the other studied solutes, which present one HBA and one HBD site, (1R)-(+)-camphor has only one HB acceptor site (from the carbonyl group). Therefore, solvents with high Hansen HB parameters, such as 1,2-propanediol, might not be as appropriate for solubilizing this monoterpene ketone.

Regarding the phenolic thymol, the highest solubility values are observed in acetonitrile, ethyl acetate, and 1,2-propanediol. In contrast, the lowest values among the organics are registered in the nonpolar hexane and R-(+)-limonene. Similar to l-(−)-menthol, the solubilities of thymol in the organic solvents are quite high, probably related to their low melting temperature and moderate melting enthalpies, and the favorable interactions in the solution.^25,37^ However, although thymol and menthol are structurally very similar, the electron resonance of thymol enhances the HBD capability (i.e., acidity) of its hydroxyl group.^58^ This leads to better thymol–solvent interactions and, thus, smaller activity coefficients than its menthol counterpart (with the exception of the fully apolar hexane and limonene). At 313.2 K, around 9 K below the melting point of thymol, its lowest observed solubility (in organic solvents) is *x* = 0.807 (in R-(+)-limonene), which is very close to the ideal solubility (*x*_ideal_ = 0.832), and higher than the solubility measured for all other monoterpenoids.

### 4.2 Comparison with Literature Data

**Figure 1:**
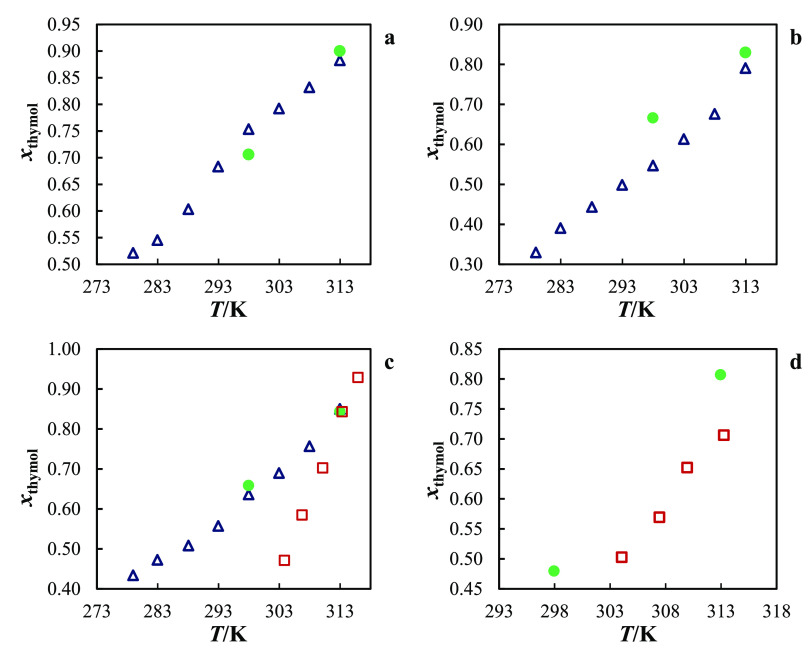
Comparison of the experimental solubility data of thymol obtained in this work and available in the literature, in different organic solvents: (a) acetonitrile; (b) 1-butanol; (c) ethanol; (d) R-(+)-limonene. Experimental data: (green circle solid) this work; (Δ) Zhu et al.;^30^ (□) Bermejo et al.^36^

Whenever possible, the experimental data obtained in this work were compared to the scarce literature data^28−30,36,59,60^ overviewed in Table S3 in the SI. The solubilities of thymol in acetonitrile, 1-butanol, ethanol, and limonene are compared in Figure 1. To the best of our knowledge, the solubilities of l-(−)-menthol in the studied organic solvents are reported here for the first time.

As can be seen in Figure 1, the solubilities of thymol in acetonitrile, 1-butanol, and ethanol from this work are in very good agreement with the data reported by Zhu et al.^30^ For these systems, an average relative deviation (calculated as the absolute difference between the solubility data obtained in this work and the literature value, divided by the literature solubility value) of 4.4% is found. For the thymol/ethanol mixture, the solubility curve reported by Bermejo et al.^36^ is considerably different from the remaining data. Regarding the thymol/R-(+)-limonene, the value obtained in this work at 313.2 K is 14% higher than the value found by Bermejo and co-authors.^36^

For borneol and camphor, a literature search showed that temperature-dependent solubility curves were only available for their racemic mixtures in ethanol.^29^ Other data are available,^28,59,60^ though the stereoisomer of the solute was not specified. As discussed by Coquerel,^61^ the solubility behavior of a specific enantiomer or a racemic mixture in a given solvent system might be quite distinct, and a direct comparison of the available solubility data obtained in this work and those reported by Chen et al.^29^ is not straightforward. Nonetheless, in those cases, the mole fraction water solubility obtained in this work for (−)-borneol (9.9 × 10^–5^) is consistent with the literature average^59,60^ of the values presented in Table S3 (8.33 × 10^–5^). Similarly, moderate relative deviations are observed between the solubilities of camphor measured in this work and those reported by Lin and Nash^28^ (at 298.2 K) in ethyl acetate (deviation = 7.5%), hexane (deviation = 18.2%), and 1,2-propylene glycol (deviation = 11.5%).

### 4.3 Abraham Solvation Model

For all of the studied solutes, the model descriptors (*E*, *S*, *A*, *B*, and *V*) have been previously reported^62−65^ and are presented in Table S4 in the SI. Nevertheless, poor descriptions of the experimental data obtained in this work were found using the descriptors available in the literature, with ARD often superior to 40% (Table S4). The isomeric form of the solute was not specified, and the available descriptors were probably regressed from partition coefficient data only since most solubility data available in the organic solvents were reported recently.^29,30,36^ Despite the recommendations in terms of the solute concentration range adequate for this model, the descriptors were regressed in this work for the solutes using a similar strategy applied in a previous work of our group.^40^

Since the Abraham solvation model was developed based on molar solubilities, the experimental mole fraction solubilities (*x*_*i*_^exp^) were converted to the molar basis (*S*_*i*_^exp^) using the expression
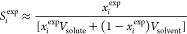
7where *V* is the molar volume (dm^3^·mol^–1^), calculated from density data collected from the literature.

The descriptors *E* and *V* were calculated using the procedure illustrated by Abraham and co-authors, while the other remaining solute parameters (*S*, *A*, and *B*) were obtained by simultaneously solving a reduced set of LFERs (eq 1) using the solubility data measured by us, at 298.2 K, in water and five organic solvents (acetonitrile, ethanol, ethyl acetate, hexane, and 1,2-propanediol). For (1R)-(+)-camphor, the hydrogen-bond acidity parameter (*A*) was set to 0 since no HB-donor site is available in its chemical structure. The estimated Abraham solute descriptors are presented in Table 3, along with the obtained ARDs and the outlier systems.

**Table 3:** Estimated Abraham Descriptors for the Monoterpenoids, Outlier Solvent, and ARD (%) Obtained Using Solubilities in Water and Five Pure Organic Solvents in the Correlation Set

compound	*E*	*S*	*A*	*B*	*V*	outlier	ARD (%)
(−)-borneol	0.688	0.589	0.104	0.672	1.359	1,2-propanediol	12
(1R)-(+)-camphor	0.570	0.654	0	0.583	1.316	acetonitrile	26
l-(−)-menthol	0.366	0.474	0	0.620	1.468	1,2-propanediol	36
thymol	0.824	0.647	0.031	0.600	1.339	1,2-propanediol	33

A global ARD of 27% was obtained in the estimation of the model descriptors, which is comparable to the results found in our previous work.^40^ The best description was obtained for (−)-borneol (ARD = 12%), while the biggest deviations are observed for l-(−)-menthol (ARD = 36%) and thymol (ARD = 33%). The latter solutes generally present higher solubilities in the studied organic solvents than the other two monoterpenoids (with only a few exceptions), while values registered for (−)-borneol are typically the lowest. This confirms that the Abraham model is more suitable for representing moderate to low solubility data values.^27^

In most cases, the estimated *E* descriptors are close to those found in the literature,^62−65^ but larger differences can be observed for *S*, *A*, and *B*, particularly for thymol, where differences around 0.2–0.4 units are observed.^63^ For the other solutes, the model descriptors obtained in this work usually deviate less than 0.2 units from the values listed in Table S4. It is relevant to mention that the descriptors reported by Abraham and co-authors^62,63^ were estimated using reduced sets of experimental partition coefficients data, which might not entirely cover the solvation behavior of the saturated conditions addressed here. On the other hand, the methodology is highly dependent on the available solubility values in water, which were updated with new information in this work.

**Figure 2:**
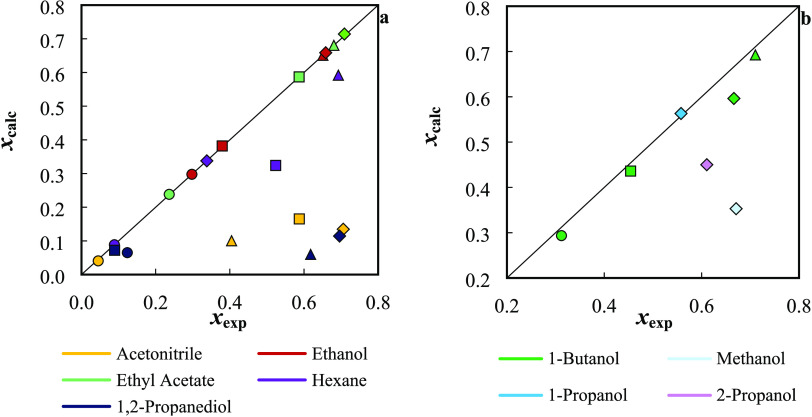
Comparison between the experimental and calculated solubility data by the Abraham solvation model, at 298.2 K: (a) correlation; (b) prediction. Filled symbols correspond to: ○, (−)-borneol; □, (1R)-(+)-camphor; Δ, l-(−)-menthol; and ◊, thymol.

The parameters presented in Table 3 were used to estimate the solubility data, at 298.2 K, of the stereoisomers studied in this work, in solvents for which the Abraham descriptors are also available (solubility of all solutes in 1-butanol measured in this work and of thymol in other pure solvents^30^). A complete overview of the correlation and predicted results is presented in Figure 2.

The highest relative deviations in the correlation step were found in 1,2-propanediol for l-(−)-menthol and thymol, but in acetonitrile, the results are also poor. Nonetheless, the model usually delivers very good solubility estimations in ethanol, ethyl acetate, and hexane. Moreover, as shown in Figure 2b, good prediction results are offered by the model with global ARDs of 8%.

### 4.4 NRTL-SAC

To the best of our knowledge, the NRTL-SAC conceptual segments for the studied monoterpenoids were not reported yet. The optimization of the solute parameters (*X*, *Y*^–^, *Y*^+^, *Z*) was carried out using the nonlinear least-squares method. The objective function to minimize was defined as the global ARD (eq 6).

From the previous experience in our group, a reduced set of experimental solubility data obtained in structurally different solvent systems is an adequate correlation database.^40^ Thus, the experimental solubilities in five solvents (water, acetonitrile, ethanol, ethyl acetate, and hexane), at 298.2 and 313.2 K, were used. After the predictive capability of the model was also checked by estimating the solubilities in 1-butanol, R-(+)-limonene, and in other solvents found in the literature.^30,36^ Again, the solubility data of the racemic mixtures,^29^ or of stereoisomers that were not identified,^28^ were not included in the predicted set due to the solubility dependency on the solute’s phase transition properties (eqs 4 and 5).

**Figure 3:**
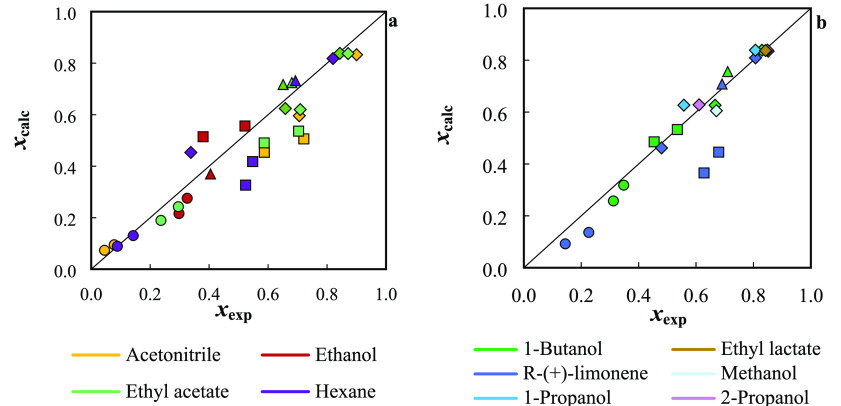
Comparison between the experimental and calculated solubility data at 298.2 and 313.2 K by the NRTL-SAC model: (a) correlation; (b) prediction. Filled symbols correspond to: ○, (−)-borneol; □, (1R)-(+)-camphor; Δ, l-(−)-menthol; and ◊, thymol.

The estimated NRTL-SAC parameters, the global ARD, and the outlier solvent for each solute are summarized in Table 4, while a visual description of the correlation and prediction results is given in Figure 3. For a better comparison, the solubilities in water were excluded from Figure 3 and are presented separately in Figure S1 in the SI. The solvent’s molecular parameters were retrieved from the literature.^32,45,50^

**Table 4:** Estimated NRTL-SAC Molecular Parameters, Outlier Solvent, and ARD (%) for the Studied Monoterpenoids

compound	*X*	*Y*^–^	*Y*^+^	*Z*	outlier	ARD (%)
(−)-borneol	0.734	0	0	0.374	acetonitrile	19
(1R)-(+)-camphor	1.324	2.223	0.189	0.089	water	27
l-(−)-menthol	0.844	0.216	0	0.054	ethanol	7
thymol	0.748	0.757	0.832	0	hexane	8

A very good description of the available solubility data for the monoterpenoids is obtained using the NRTL-SAC model, with global ARDs obtained in the correlation (15%) and prediction (14%). Excellent results were obtained for l-(−)-menthol and thymol (ARD lower than 8%) during correlation, and even lower for the predictions, where ARD is close to 4% for both solutes. In the case of (−)-borneol, ARDs of 19 and 26% were obtained in the correlation and prediction, respectively.

Among the four monoterpenoids, the overall weakest performance is for (1R)-(+)-camphor, with an ARD of 27 and 21% for correlation and prediction, respectively. Although these deviations are superior to those obtained for all other systems, they are in line with or better than those reported in previous works for other biomolecules.^40,46,49,66,67^ Particularly for water, the NRTL-SAC is not capable of predicting the reduction in the solubility of camphor as the temperature rises from 298.2 to 313.2 K (shown in Table 2), providing a considerably lower solubility estimate (*x*_calc_ = 3.9 × 10^–5^) than the experimental value (*x*_exp_ = 1.4 × 10^–4^) at 298.2 K.^24^ Notably, for such small solubility values, the model delivers very reliable water solubility estimations for all other monoterpenoids, as illustrated in Figure S1, with a global ARD of 11% considering the four solutes.

### 4.5 COSMO-RS

**Figure 4:**
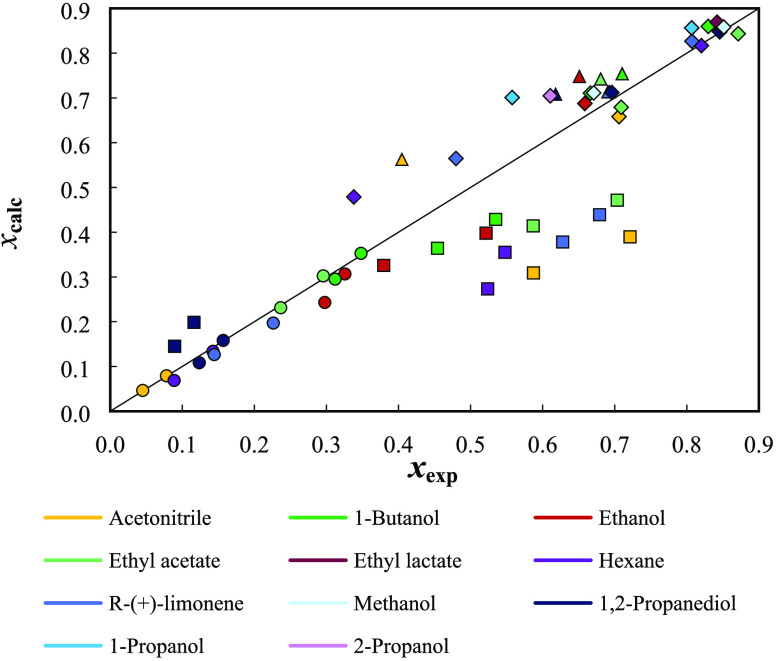
Overview of the experimental and calculated solubility data (at 298.2 ±1 and 313.2 ±1 K) by the COSMO-RS model. Filled symbols correspond to: ○, (−)-borneol; □, (1R)-(+)-camphor; Δ, l-(−)-menthol; and ◊, thymol.

In Figure 4, the experimental solubilities of the monoterpenoids in organic solvents compiled in this work^30,36,37^ are compared with the values obtained with COSMO-RS. The COSMO-RS model delivers a very good representation of the solubility data of the monoterpenoids in organic solvents, showing a global ARD of 16%. High-quality predictions were obtained for thymol (ARD = 7%), (−)-borneol (ARD = 8%), and l-(−)-menthol (ARD = 13%). For these solutes, worse results were observed for l-(−)-menthol + acetonitrile and thymol/(−)-borneol + hexane. In particular for thymol, a remarkably low ARD of 2.5% is observed for the available solubility data at 313.2 K, showing that COSMO-RS can deliver reliable predictions considering the solubility change with temperature.

Concerning the solubility of (1R)-(+)-camphor in organic solvents, the calculated global ARD is 37%. In Figure 4, the systematic underestimation of (1R)-(+)-camphor solubility for most solvents is notable, which is very significant in organic solvents of different characteristics, namely, acetonitrile, hexane, limonene, and ethyl acetate. Nonetheless, the (1R)-(+)-camphor predicted solubilities in alcohols are satisfactory (ARD < 25%).

The experimental and predicted solubilities in water are depicted in Figure S2 in the SI. For these systems, the best solubility description is achieved for l-(−)-menthol, followed by thymol, (−)-borneol, and (1R)-(+)-camphor. The values are now overestimated, and excepting (1R)-(+)-camphor, the model provides water solubilities of the same order of magnitude as the experimental ones. As discussed in our previous work,^24^ the calculation of monoterpenoids solubility in aqueous systems, using either semiempirical or predictive models, is a challenging task. Nevertheless, the predictive COSMO-RS often provides better performances than other more empirical options.

**Figure 5:**
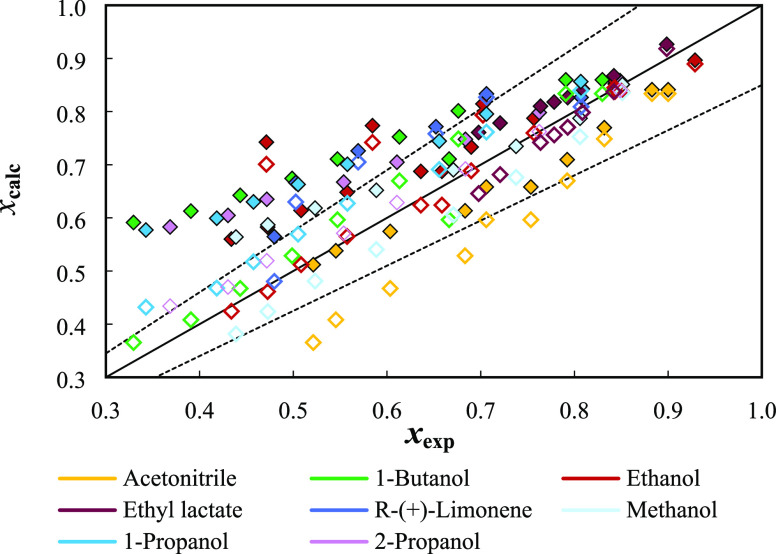
Comparison of the temperature-dependent experimental and calculated solubilities of thymol by the COSMO-RS model (filled symbols) and the NRTL-SAC model (empty symbols). The dashed lines correspond to *x* ± 0.15*x*.

Since appropriate descriptions of the solubility data at 298.2 and 313.2 K were obtained with both NRTL-SAC and COSMO-RS models, they were tested to represent the temperature solubility curves of thymol using data available in the literature^30,36,37^ and measured in this work. The temperature solubility curves are depicted in Figure S3 in the SI, while a comparison of the experimental and calculated solubilities is presented in Figure 5.

In general, both models offer a qualitative picture of the solubility curves, with NRTL-SAC (ARD = 9%) performing better than COSMO-RS (ARD = 17%). Two lines representing ARD limits of 15% were included in Figure 5. As can be seen, much of the values are inside that region, being the alcohols 1-propanol, 2-propanol, and 1-butanol the outliers for COSMO-RS, while acetonitrile is the solvent presenting most of the data points out of the delimited region. When comparing both models, the NRTL-SAC depends on the availability of the model parameters for the solute and the solvent, limiting its application for systems where this information is known. On the other hand, COSMO-RS presents a full predictive character, capable of providing solubility curves only from the chemical structures of the individual compounds in a mixture. Therefore, the ability of the COSMO-RS model to deliver solubility estimations suggests its application in the preliminary solvent screening of different solutes, particularly when little experimental information is available.

## 5 Conclusions

New experimental solubility data of three monoterpenoids ((−)-borneol, (1R)-(+)-camphor, and thymol) in acetonitrile, 1-butanol, ethanol, ethyl acetate, hexane, R-(+)-limonene, and 1,2-propanediol, at 298.2 and 313.2 K, are reported. For (−)-borneol, experimental water solubilities at 298.2 and 312.2 K were also measured. The solubilities of l-(−)-menthol in the same set of organic solvents were investigated at 298.2 K. The Abraham solvation and NRTL-SAC models were successfully applied to describe the solubility data measured here, and most data were retrieved from the open literature. In the case of the Abraham model, global ARD of 27 and 8% were obtained in the correlation and prediction steps, respectively, while the lowest global deviations were observed for camphor and borneol. Regarding the NRTL-SAC model, the ARD for the solutes varied between 7 and 27% in the correlation and between 4 and 26% in the prediction, considering the available solubility at 298.2 and 313.2 K, being the best performances found for thymol and l-(−)-menthol (ARDs = 6%). Nevertheless, the use of these models depends on the availability of the solute parameters or a small set of experimental data to estimate them. Moreover, the absence of parameters for some solvents (1,2-propanediol for the NRTL-SAC model, and ethyl lactate and limonene for the Abraham solvation model) precludes covering all of the solubility data.

On the other hand, COSMO-RS offers a good representation of the solubility data in organic solvents at 298.2 and 313.2 K, presenting ARDs between 7% (thymol) and 37% ((1R)-(+)-camphor), and a global ARD of 16%. The overall performance of COSMO-RS in describing the solubilities of the monoterpenoids was comparable to those registered by other hybrid approaches, which is a remarkable achievement of this fully predictive model that requires only structural information from the components present in the mixture.

## Supporting Information Available

Analytical procedure for quantification of experimental solubility data by UV–vis spectroscopy and gas chromatography; experimental water solubilities of the solutes at 298.2 and 313.2 K; experimental activity coefficients for the solutes in water and organic solvents at 298.2 and 313.2 K; overview of the available solubility data for the binary systems under study; list of the Abraham descriptors for the solutes found in the literature; and comparison of the experimental temperature-dependent solubility curves with those predicted by the NRTL-SAC and COSMO-RS models (PDF)

## References

[ref1] Zielińska-Błajet M.; Feder-Kubis J. Monoterpenes and Their Derivatives—Recent Development in Biological and Medical Applications. Int. J. Mol. Sci. 2020, 21, 1–38. 10.3390/ijms21197078. PMC7582973 32992914

[ref2] Tchimene M. K.; Okunji C. O.; Iwu M. M.; Kuete V. Monoterpenes and Related Compounds from the Medicinal Plants of Africa. In Medicinal Plant Research in Africa, Kuete V., Ed.; Elsevier: Nsukka, 2013; pp 1–32.

[ref3] Sharmeen J. B.; Mahomoodally F. M.; Zengin G.; Maggi F. Essential Oils as Natural Sources of Fragrance Compounds for Cosmetics and Cosmeceuticals. Molecules 2021, 26, 666 10.3390/molecules26030666. 33514008 PMC7865210

[ref4] Perri F.; Coricello A.; Adams J. D. Monoterpenoids: The next Frontier in the Treatment of Chronic Pain?. J — Multidiscip. Sci. J. 2020, 3, 195–214. 10.3390/j3020016.

[ref5] Kabir A.; Cacciagrano F.; Tartaglia A.; Lipsi M.; Ulusoy H. I.; Locatelli M. Analysis of Monoterpenes and Monoterpenoids. In Recent Advances in Natural Products Analysis, Nabavi S. M.; Saeedi M.; Nabavi S. F.; Silva A. S., Eds.; Elsevier, 2020; pp 274–286.

[ref6] Hamidpour R.; Hamidpour S.; Hamidpour M.; Shahlari M. Camphor (*Cinnamomum Camphora*), a Traditional Remedy with the History of Treating Several Diseases. Int. J. Case Rep. Images 2013, 4, 86–88. 10.5348/ijcri-2013-02-267-RA-1.

[ref7] Chen W.; Vermaak I.; Viljoen A. Camphor-A Fumigant during the Black Death and a Coveted Fragrant Wood in Ancient Egypt and Babylon-A Review. Molecules 2013, 18, 5434–5454. 10.3390/molecules18055434. 23666009 PMC6270224

[ref8] Bhatia S. P.; Letizia C. S.; Api A. M. Fragrance Material Review on Borneol. Food Chem. Toxicol. 2008, 46, S77–S80. 10.1016/j.fct.2008.06.031. 18640181

[ref9] Liu S.; Long Y.; Yu S.; Zhang D.; Yang Q.; Ci Z.; Cui M.; Zhang Y.; Wan J.; Li D.; Shi A.; Li N.; Yang M.; Lin J. Borneol in Cardio-Cerebrovascular Diseases: Pharmacological Actions, Mechanisms, and Therapeutics. Pharmacol. Res. 2021, 169, 105627 10.1016/j.phrs.2021.105627. 33892091

[ref10] Zhang Q.-L.; Fu B. M.; Zhang Z.-J. Borneol, a Novel Agent That Improves Central Nervous System Drug Delivery by Enhancing Blood–Brain Barrier Permeability. Drug Delivery 2017, 24, 1037–1044. 10.1080/10717544.2017.1346002. 28687052 PMC8241164

[ref11] Kulkarni M.; Sawant N.; Kolapkar A.; Huprikar A.; Desai N. Borneol: A Promising Monoterpenoid in Enhancing Drug Delivery across Various Physiological Barriers. AAPS PharmSciTech 2021, 22, 145 10.1208/s12249-021-01999-8. 33913042

[ref12] Bhatia S. P.; MCGinty D.; Letizia C. S.; Api A. M. Fragrance Material Review on Menthol. Food Chem. Toxicol. 2008, 46, S209–S214. 10.1016/j.fct.2008.06.059. 18640186

[ref13] A Farco J.; Grundmann O. Menthol - Pharmacology of an Important Naturally Medicinal “Cool. Mini Rev. Med. Chem. 2013, 13, 124–131. 10.2174/138955713804484686. 23061635

[ref14] Harris B. Menthol: A Review of Its Thermoreceptor Interactions and Their Therapeutic Applications. Int. J. Aromather. 2006, 16, 117–131. 10.1016/j.ijat.2006.09.010.

[ref15] Makoś P.; Słupek E.; Gębicki J. Hydrophobic Deep Eutectic Solvents in Microextraction Techniques–A Review. Microchem. J. 2020, 152, 104384 10.1016/j.microc.2019.104384.

[ref16] Rahman M. S.; Roy R.; Jadhav B.; Hossain M. N.; Halim M. A.; Raynie D. E. Formulation, Structure, and Applications of Therapeutic and Amino Acid-Based Deep Eutectic Solvents: An Overview. J. Mol. Liq. 2021, 321, 114745 10.1016/j.molliq.2020.114745.

[ref17] Dwamena A. Recent Advances in Hydrophobic Deep Eutectic Solvents for Extraction. Separations 2019, 6, 9 10.3390/separations6010009.

[ref18] Escobar A.; Pérez M.; Romanelli G.; Blustein G. Thymol Bioactivity: A Review Focusing on Practical Applications. Arab. J. Chem. 2020, 13, 9243–9269. 10.1016/j.arabjc.2020.11.009.

[ref19] Jyoti; Dheer D.; Singh D.; Kumar G.; Karnatak M.; Chandra S.; Prakash Verma V.; Shankar R. Thymol Chemistry: A Medicinal Toolbox. Curr. Bioact. Compd. 2019, 15, 454–474. 10.2174/1573407214666180503120222.

[ref20] Najafloo R.; Behyari M.; Imani R.; Nour S. A Mini-Review of Thymol Incorporated Materials: Applications in Antibacterial Wound Dressing. J. Drug Delivery Sci. Technol. 2020, 60, 101904 10.1016/j.jddst.2020.101904.

[ref21] Costa M. F.; Durço A. O.; Rabelo T. K.; Barreto R.; de S. S.; Guimarães A. G. Effects of Carvacrol, Thymol and Essential Oils Containing Such Monoterpenes on Wound Healing: A Systematic Review. J. Pharm. Pharmacol. 2019, 71, 141–155. 10.1111/jphp.13054. 30537169

[ref22] Alagawany M.; Farag M. R.; Abdelnour S. A.; Elnesr S. S. A Review on the Beneficial Effect of Thymol on Health and Production of Fish. Rev. Aquac. 2021, 13, 632–641. 10.1111/raq.12490.

[ref23] Martins M. A. R.; Silva L. P.; Ferreira O.; Schröder B.; Coutinho J. A. P.; Pinho S. P. Terpenes Solubility in Water and Their Environmental Distribution. J. Mol. Liq. 2017, 241, 996–1002. 10.1016/j.molliq.2017.06.099.

[ref24] Vilas-Boas S. M.; da Costa M. C.; Coutinho J. A. P.; Ferreira O.; Pinho S. P. Octanol–Water Partition Coefficients and Aqueous Solubility Data of Monoterpenoids: Experimental, Modeling, and Environmental Distribution. Ind. Eng. Chem. Res. 2022, 61, 3154–3167. 10.1021/acs.iecr.1c04196.

[ref25] Štejfa V.; Bazyleva A.; Fulem M.; Rohlíček J.; Skořepová E.; Růžička K.; Blokhin A. V. Polymorphism and Thermophysical Properties of L- and DL-Menthol. J. Chem. Thermodyn. 2019, 131, 524–543. 10.1016/j.jct.2018.11.004. PMC7067000 32165766

[ref26] Abraham M. H.; Ibrahim A.; Zissimos A. M. Determination of Sets of Solute Descriptors from Chromatographic Measurements. J. Chromatogr. A 2004, 1037, 29–47. 10.1016/j.chroma.2003.12.004. 15214659

[ref27] Abraham M. H.; Smith R. E.; Luchtefeld R.; Boorem A. J.; Luo R.; Jr W. E. A. Prediction of Solubility of Drugs and Other Compounds in Organic Solvents. J. Pharm. Sci. 2010, 99, 1500–1515. 10.1002/jps.21922. 19774653

[ref28] Lin H. -M.; Nash R. A. An Experimental Method for Determining the Hildebrand Solubility Parameter of Organic Nonelectrolytes. J. Pharm. Sci. 1993, 82, 1018–1026. 10.1002/jps.2600821001. 8254486

[ref29] Chen J.; He J.; Li N.; Zheng H.; Zhao S. Determination and Correlation of Solubility of Borneol, Camphor, and Isoborneol in Different Solvents. J. Chem. Eng. Data 2019, 64, 1826–1833. 10.1021/acs.jced.9b00045.

[ref30] Zhu P.; Chen Y.; Fang J.; Wang Z.; Xie C.; Hou B.; Chen W.; Xu F. Solubility and Solution Thermodynamics of Thymol in Six Pure Organic Solvents. J. Chem. Thermodyn. 2016, 92, 198–206. 10.1016/j.jct.2015.09.010.

[ref31] Chen C.-C.; Song Y. Solubility Modeling with NRTL Segment Activity Coefficient Model. Ind. Eng. Chem. Res 2004, 43, 8354–8362. 10.1021/ie049463u.

[ref32] Chen C.-C.; Crafts P. A. Correlation and Prediction of Drug Molecule Solubility with the NRTL-SAC Model. In Computer Aided Chemical Engineering, 2006; Vol. 21, pp 859–864.

[ref33] Klamt A. Conductor-like Screening Model for Real Solvents: A New Approach to the Quantitative Calculation of Solvation Phenomena. J. Phys. Chem. A 1995, 99, 2224–2235. 10.1021/j100007a062.

[ref34] Klamt A.; Jonas V.; Bürger T.; Lohrenz J. C. W. Refinement and Parametrization of COSMO-RS. J. Phys. Chem. A 1998, 102, 5074–5085. 10.1021/jp980017s.

[ref35] Eckert F.; Klamt A. Fast Solvent Screening via Quantum Chemistry: COSMO-RS Approach. AIChE J. 2002, 48, 369–385. 10.1002/aic.690480220.

[ref36] Villanueva Bermejo D.; Angelov I.; Vicente G.; Stateva R. P.; Rodriguez García-Risco M.; Reglero G.; Ibañez E.; Fornari T. Extraction of Thymol from Different Varieties of Thyme Plants Using Green Solvents. J. Sci. Food Agric. 2015, 95, 2901–2907. 10.1002/jsfa.7031. 25445203

[ref37] Manic M. S.; Villanueva D.; Fornari T.; Queimada A. J.; Macedo E. A.; Najdanovic-Visak V. Solubility of High-Value Compounds in Ethyl Lactate: Measurements and Modeling. J. Chem. Thermodyn. 2012, 48, 93–100. 10.1016/j.jct.2011.12.005.

[ref38] Štejfa V.; Fulem M.; Růžička K.; Červinka C. Thermodynamic Study of Selected Monoterpenes II. J. Chem. Thermodyn. 2014, 79, 272–279. 10.1016/j.jct.2013.12.012.

[ref39] Rietveld I. B.; Barrio M.; Veglio N.; Espeau P.; Tamarit J. L.; Céolin R. Temperature and Composition-Dependent Properties of the Two-Component System d- and l-Camphor at “ordinary” Pressure. Thermochim. Acta 2010, 511, 43–50. 10.1016/j.tca.2010.07.023.

[ref40] Vilas-Boas S. M.; Alves R. S.; Brandão P.; Campos L. M. A.; Coutinho J. A. P.; Pinho S. P.; Ferreira O. Solid-Liquid Phase Equilibrium of Trans-Cinnamic Acid, p-Coumaric Acid and Ferulic Acid in Water and Organic Solvents: Experimental and Modelling Studies. Fluid Phase Equilib. 2020, 521, 112747 10.1016/j.fluid.2020.112747.

[ref41] Bradley A. E.; Hardacre C.; Holbrey J. D.; Johnston S.; McMath S. E. J.; Nieuwenhuyzent M. Small-Angle x-Ray Scattering Studies of Liquid Crystalline 1-Alkyl-3-Methylimidazolium Salts. Chem. Mater. 2002, 14, 629–635. 10.1021/cm010542v.

[ref42] Stovall D. M.; Dai C.; Zhang S.; Acree W. E.; Abraham M. H. Abraham Model Correlations for Describing Solute Transfer into Anhydrous 1,2-Propylene Glycol for Neutral and Ionic Species. Phys. Chem. Liq. 2016, 54, 1–13. 10.1080/00319104.2015.1058379.

[ref43] Abraham M. H.; McGowan J. C. The Use of Characteristic Volumes to Measure Cavity Terms in Reversed Phase Liquid Chromatography. Chromatographia 1987, 23, 243–246. 10.1007/BF02311772.

[ref44] Chen C. C.; Simoni L. D.; Brennecke J. F.; Stadtherr M. A. Correlation and Prediction of Phase Behavior of Organic Compounds in Ionic Liquids Using the Nonrandom Two-Liquid Segment Activity Coefficient Model. Ind. Eng. Chem. Res. 2008, 47, 7081–7093. 10.1021/ie800048d.

[ref45] Aguda R.; Chen C.-C. Solubility of Nutraceutical Compounds in Generally Recognized as Safe Solvents at 298 K. Int. J. Chem. Eng. Appl. 2016, 7, 289–294. 10.18178/ijcea.2016.7.5.591.

[ref46] Mota F. L.; Carneiro A. P.; Queimada A. J.; Pinho S. P.; Macedo E. A. Temperature and Solvent Effects in the Solubility of Some Pharmaceutical Compounds: Measurements and Modeling. Eur. J. Pharm. Sci. 2009, 37, 499–507. 10.1016/j.ejps.2009.04.009. 19406228

[ref47] Bouillot B.; Teychené S.; Biscans B. An Evaluation of Thermodynamic Models for the Prediction of Drug and Drug-like Molecule Solubility in Organic Solvents. Fluid Phase Equilib. 2011, 309, 36–52. 10.1016/j.fluid.2011.06.032.

[ref48] Widenski D. J.; Abbas A.; Romagnoli J. A. Comparison of Different Solubility Equations for Modeling in Cooling Crystallization. Chem. Eng. Process. Process Intensif. 2010, 49, 1284–1297. 10.1016/j.cep.2010.09.018.

[ref49] Sheikholeslamzadeh E.; Rohani S. Solubility Prediction of Pharmaceutical and Chemical Compounds in Pure and Mixed Solvents Using Predictive Models. Ind. Eng. Chem. Res. 2012, 51, 464–473. 10.1021/ie201344k.

[ref50] Vilas-Boas S. M.; Cordova I. W.; Kurnia K. A.; Almeida H. H. S.; Gaschi P. S.; Coutinho J. A. P.; Pinho S. P.; Ferreira O. Comparison of Two Computational Methods for Solvent Screening in Countercurrent and Centrifugal Partition Chromatography. J. Chromatogr. A 2022, 1666, 462859 10.1016/j.chroma.2022.462859. 35124362

[ref51] Prausnitz J. M.; Lichtenthaler R. N.; de Azevedo E. G. Molecular Thermodynamics of Fluid-Phase Equilibria, Vol. 3; Pretnice Hall PTR, 1999; Vol. 3.

[ref52] Weimer R. F.; Prausnitz J. M. Complex Formation between Carbon Tetrachloride and Aromatic Hydrocarbons. J. Chem. Phys. 1965, 42, 3643–3644. 10.1063/1.1695773.

[ref53] Choi P. B.; Mclaughlin E. Effect of a Phase Transition on the Solubility of a Solid. AIChE J. 1983, 29, 150–153. 10.1002/aic.690290121.

[ref54] BIOVIA COSMOtherm. Release 2021, Dassault Systèmes, 2021.

[ref55] Steffen C.; Thomas K.; Huniar U.; Hellweg A.; Rubner O.; Schroer A. Software News and Updates TmoleX — A Graphical User Interface for TURBOMOLE. J. Comput. Chem. 2010, 31, 2967–2970. 20928852 10.1002/jcc.21576

[ref56] BIOVIA COSMOtherm 2020. Reference Manual; 2020.

[ref57] Hansen C. M. Hansen Solubility Parameters: A User’s Handbook, 2nd ed.; CRC Press, 2007.

[ref58] Abranches D. O.; Martins M. A. R.; Silva L. P.; Schaeffer N.; Pinho S. P.; Coutinho J. A. P. Phenolic Hydrogen Bond Donors in the Formation of Non-Ionic Deep Eutectic Solvents: The Quest for Type V DES. Chem. Commun. 2019, 55, 10253–10256. 10.1039/C9CC04846D. 31393483

[ref59] Knobloch K.; Pauli A.; Iberl B.; Weigand H.; Weis N. Antibacterial and Antifungal Properties of Essential Oil Components. J. Essent. Oil Res. 1989, 1, 119–128. 10.1080/10412905.1989.9697767.

[ref60] Mitchell S. CLXXI.—A Method for Determining the Solubility of Sparingly Soluble Substances. J. Chem. Soc. 1926, 129, 1333–1336. 10.1039/JR9262901333.

[ref61] Coquerel G. Solubility of Chiral Species as Function of the Enantiomeric Excess. J. Pharm. Pharmacol. 2015, 67, 869–878. 10.1111/jphp.12395. 25918978

[ref62] Abraham M. H.; Kumarsingh R.; Cometto-Muñiz J. E.; Cain W. S.; Rosés M.; Bosch E.; Díaz M. L. The Determination of Solvation Descriptors for Terpenes, and the Prediction of Nasal Pungency Thresholds. J. Chem. Soc. Perkin Trans. 2 1998, 11, 2405–2411. 10.1039/a805665j.

[ref63] Abraham M. H.; Acree W. E. Solute Descriptors for Phenoxide Anions and Their Use to Establish Correlations of Rates of Reaction of Anions with Iodomethane. J. Org. Chem. 2010, 75, 3021–3026. 10.1021/jo100292j. 20380416

[ref64] Karunasekara T.; Poole C. F. Determination of Descriptors for Fragrance Compounds by Gas Chromatography and Liquid-Liquid Partition. J. Chromatogr. A 2012, 1235, 159–165. 10.1016/j.chroma.2012.02.043. 22405539

[ref65] Poole C. F. Wayne State University Experimental Descriptor Database for Use with the Solvation Parameter Model. J. Chromatogr. A 2020, 1617, 460841 10.1016/j.chroma.2019.460841. 31954542

[ref66] Mota F. L.; Queimada A. J.; Andreatta A. E.; Pinho S. P.; Macedo E. A. Calculation of Drug-like Molecules Solubility Using Predictive Activity Coefficient Models. Fluid Phase Equilib. 2012, 322–323, 48–55. 10.1016/j.fluid.2012.02.003.

[ref67] Tang W.; Wang Z.; Feng Y.; Xie C.; Wang J.; Yang C.; Gong J. Experimental Determination and Computational Prediction of Androstenedione Solubility in Alcohol + Water Mixtures. Ind. Eng. Chem. Res. 2014, 53, 11538–11549. 10.1021/ie501221x.

