# Supplementary material for: Modeling the Solubility of Monoterpenoids with Hybrid and Predictive Thermodynamic Tools
Source: Ind Eng Chem Res. 2023 Mar 13;62(12):5326–35. doi: 10.1021/acs.iecr.2c03991 (PMC10064338; doi:10.1021/acs.iecr.2c03991)
Supplement: Supplementary file 1 — ie2c03991_si_001.pdf [file ie2c03991_si_001.pdf]

Supporting Information

Modeling the solubility of monoterpenoids with hybrid and predictive thermodynamic tools

Sérgio M. Vilas-Boas[a,b,c], Isabella W. Cordova[a,b,c], Dinis O. Abranches[c], João A.P. Coutinho[c], Olga Ferreira[a,b*], Simão P. Pinho[a,b*]

[a]Centro de Investigação de Montanha (CIMO), Instituto Politécnico de Bragança, Campus de Santa Apolónia, 5300-253 Bragança, Portugal

[b]Laboratório para a Sustentabilidade e Tecnologia em Regiões de Montanha, Instituto Politécnico de Bragança, Campus de Santa Apolónia, 5300-253 Bragança, Portugal

[c]CICECO − Aveiro Institute of Materials, Department of Chemistry, University of Aveiro, 3810-193 Aveiro, Portugal.

*Corresponding authors:

Olga Ferreira, Tel.: +351273303087, E-mail: oferreira@ipb.pt

Simão P. Pinho, Tel.: +351273303086, E-mail: spinho@ipb.pt

Section S1 – Experimental

Analytical procedures for the solubility experiments

The solubilities of (1R)-(+)-camphor and thymol in the studied organic solvents were determined by UV-vis spectroscopy (T70, PG Instruments) at 287 nm and 275.5 nm, respectively. Calibration curves ($R^2 > 0.999$) were built using at least seven standards independently prepared using ethanol/water (50:50 in mass proportion) as the solvent. At least two independent readings were carried out for each sample collected from the saturated solutions.

For (-)-borneol and L-(-)-menthol, the solubilities were quantified by gas chromatography using the internal standard method (with linalool as the internal standard and acetonitrile as the solvent). The GC (Varian, CP3800) was equipped with a split/splitless injection unit and a flame-ionization detector (FID), and fitted with a SUPELCOWAX® 10 capillary column (L x I.D. 30 m x 0.25 mm, df 0.25 μm) coupled to a deactivated fused silica pre-column (L x I.D. 30 m x 0.25 mm). The GC operating conditions were performed using the following parameters: injector and detector were kept at 523.2 K; helium was used as the carrier gas at a flow rate of 1.2 $ml \cdot min^{-1}$; the injection volume was 1 μl with a split ration of 1:60; the oven temperature was initially maintained at 393.2 K for 20-25 min, followed by a temperature ramp of 50 $K \cdot min^{-1}$ up to 503.2 K, and an isothermal step at 503.2 K for 5 min. Calibrations curves ($R^2 > 0.9999$) for each solute were built using at least seven independent standards.

Section S2 – Results and discussion

Table S1. Experimental solubilities (in mole fraction) of the monoterpenoids in water measured at 298.2 K and 313.2 K.

Monoterpenoid	$10^5\ x_{terpene}$		source
	298.2 K	313.2 K	
(–)-borneol	9.909 ± 0.366	10.722 ± 0.100	this work
(1R)-(+)-camphor	13.65	4.371	[1]
L-(–)-menthol	4.419		[1]
thymol	11.80	16.89	[2]

Table S2. Experimental activity coefficients (γ_i) of the monoterpenoids in water and organic solvents at 298.2 K and 313.2 K

Solvent	(–)-borneol		(1R)-(+)-camphor		L-(–)-menthol	thymol	
	298.2 K	313.2 K	298.2 K	313.2 K	298.2 K	298.2 K	313.2 K
acetonitrile	6.03	4.27	0.72	0.66	1.83	0.84	0.92
1-butanol	0.87	0.96	0.93	0.89	1.04	0.89	1.00
ethanol	0.91	1.02	1.11	0.92	1.14	0.90	0.99
ethyl acetate	1.15	1.13	0.72	0.68	1.09	0.84	0.96
hexane	3.07	2.34	0.81	0.87	1.07	1.76	1.01
R-(+)-limonene	1.89	1.47	0.67	0.70	1.07	1.24	1.03
1,2-propanediol	2.20	2.13	4.72	4.11	1.20	0.85	0.98
water	2746	3109	3093	10928	16757	5039	4927

Table S3. Overview of the available solubility data of the monoterpenoids for the binary systems addressed in this work.

Solute	Solvent	Temperature range (K)	Reference
(±)-borneol	Ethanol	297.1 – 348.7	[3]
(–)-borneol		298.2 – 313.2	this work
borneol[b]	water	298.2	[4c]
borneol[b]		288.2 -298.2	[5]
(–)-borneol		298.2 -313.2	this work
(±)-camphor	ethanol	303.2 – 343.2	[3]
(1R)-(+)-camphor		298.2 – 313.2	this work
camphor[b]	ethyl acetate	298.2	[6]
(1R)-(+)-camphor		298.2 – 313.2	this work
camphor[b]	hexane	298.2	[6]
(1R)-(+)-camphor		298.2 – 313.2	this work
camphor[b]	1,2-propanediol	298.2	[6]
(1R)-(+)-camphor		298.2 -313.2	this work
thymol	acetonitrile	279.2 – 313.2	[7]
		298.2 – 313.2	this work
	1-butanol	279.2 – 313.2	[7]
		298.2 – 313.2	this work
	ethanol	279.2 – 313.2	[7]
		304.1 – 316.2	[8]
		298.2 – 313.2	this work
	R(+)-limonene	304.1 – 316.2	[8]
		298.15 -313.15	this work

[a] Number of experimental data points. [b]No information regarding the main isomeric form. [c]The solubility data from the literature were converted from the original unit (volume basis) to g/100 moles of solvent.

Table S4. Abraham descriptors for the studied monoterpenoids available in literature and the obtained ARD (%) in the description of the solubility data measured in this work, at 298.2 K.

Compound	*E*	*S*	*A*	*B*	*V*	ARD(%)	Reference
borneol	0.510	0.520	0.280	0.680	1.3591	31	[9]
	0.757	0.714	0.158	0.653	1.3591	19	[10]
	0.659	0.635	0.203	0.632	1.3591	40	[11]
camphor	0.500	0.690	0	0.710	1.3161	81	[9]
	0.506	0.829	0	0.671	1.3161	79	[10]
	0.579	0.850	0	0.642	1.3161	72	[11]
menthol	0.400	0.500	0.230	0.580	1.4677	65[a]	[9]
thymol	0.822	0.840	0.440	0.430	1.3387	54[b]	[12]

[a]The solubilities in 1-butanol and ethanol were excluded from the predicted set since $x_i^{calc} > 1$ was found for those systems.

[b]The solubilities in 1-butanol, ethanol and ethyl acetate were excluded from the predicted set since $x_i^{calc} > 1$ was found for those systems.

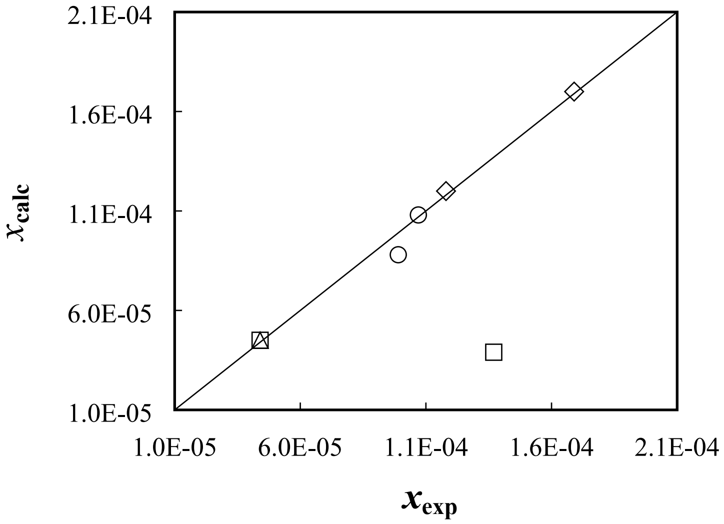

Figure S1. Comparison of the mole fraction water-solubilities calculated by NRTL-SAC with the experimental values.[1,2] Symbols correspond to: ○ - borneol, □ - camphor, △ - L-(–)-menthol, and ◇ - thymol.

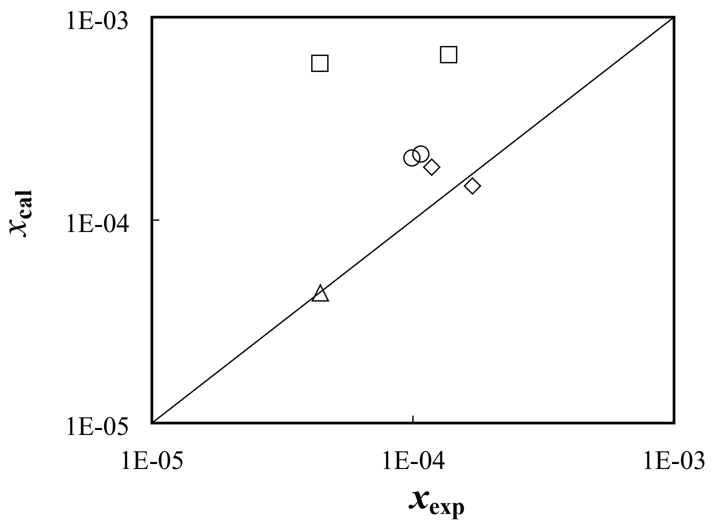

Figure S2. Comparison of the mole fraction water-solubilities calculated by COSMO-RS with the experimental values.[1,2] Symbols correspond to: ○ - borneol, □ - camphor, △ - L-(–)-menthol, and ◇ - thymol.

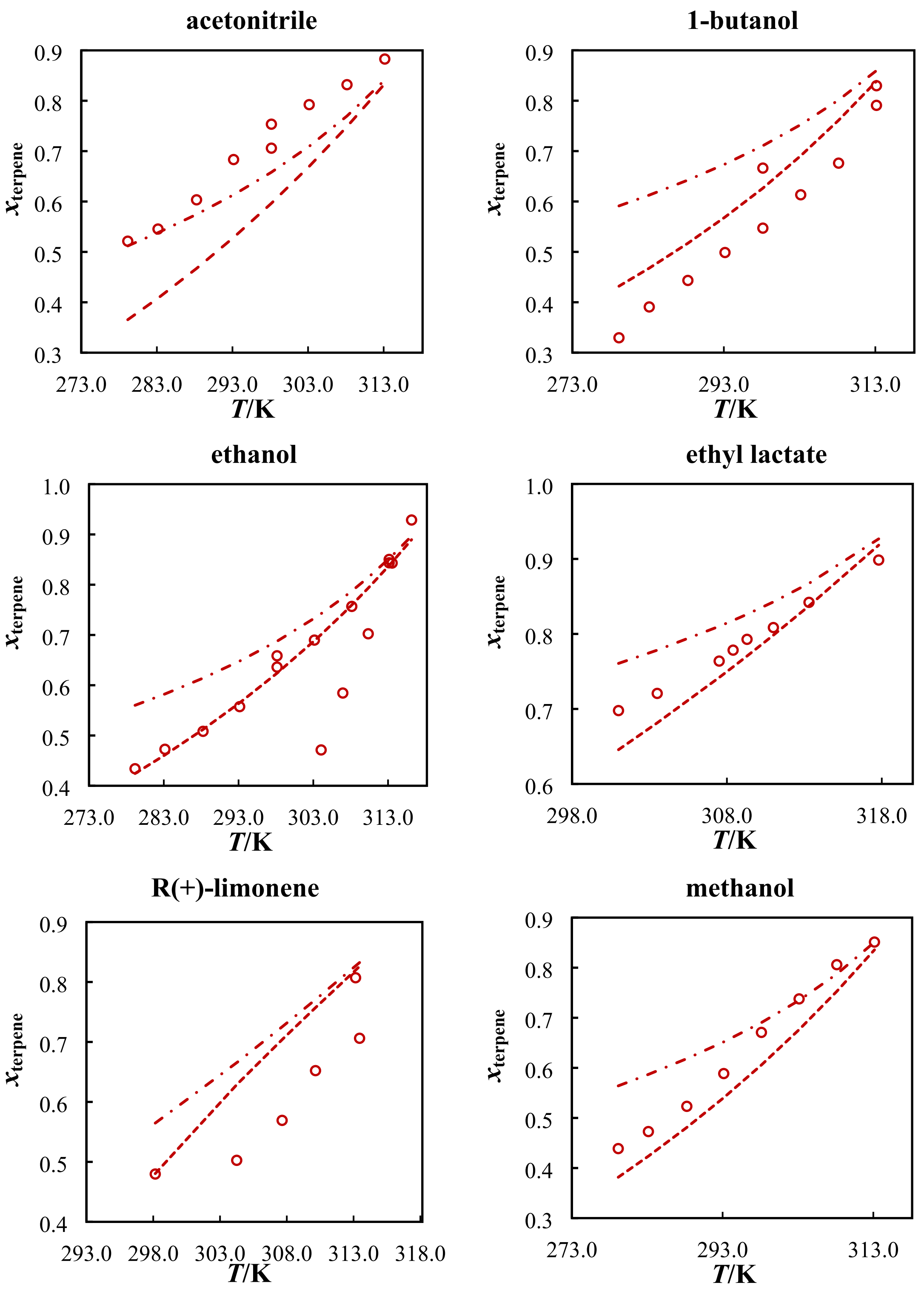
acetonitrile
1-butanol
ethanol
ethyl lactate
R(+)-limonene
methanol
$x_{terpene}$
T/K
0.3
0.4
0.5
0.6
0.7
0.8
0.9
1.0
273.0
283.0
293.0
298.0
303.0
308.0
313.0
318.0

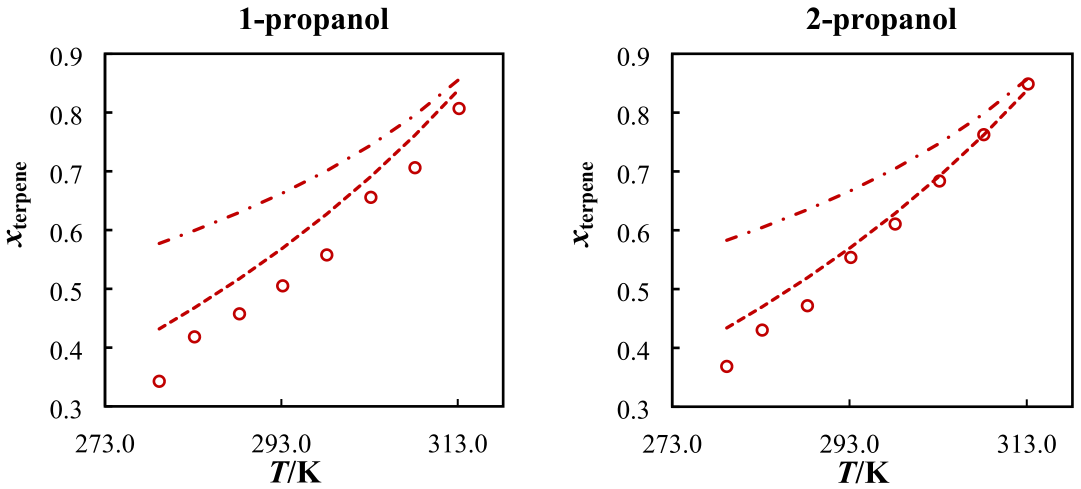

Figure S3. Comparison of the calculated solubility curves of thymol with the experimental data measured in this work and reported in literature.[7,8,13] Circles (○) correspond to the experimental data, dashed lines represent the NRTL-SAC predictions, and the dot-dash line represented the COSMO-RS predictions.

References

(1) Vilas-Boas, S. M.; da Costa, M. C.; Coutinho, J. A. P.; Ferreira, O.; Pinho, S. P. Octanol–Water Partition Coefficients and Aqueous Solubility Data of Monoterpenoids: Experimental, Modeling, and Environmental Distribution. *Ind. Eng. Chem. Res.* **2022**, *61*, 3154−3167.

(2) Martins, M. A. R.; Silva, L. P.; Ferreira, O.; Schröder, B.; Coutinho, J. A. P.; Pinho, S. P. Terpenes Solubility in Water and Their Environmental Distribution. *J. Mol. Liq.* **2017**, *241*, 996–1002.

(3) Chen, J.; He, J.; Li, N.; Zheng, H.; Zhao, S. Determination and Correlation of Solubility of Borneol, Camphor, and Isoborneol in Different Solvents. *J. Chem. Eng. Data* **2019**, *64* (4), 1826–1833.

(4) Knobloch, K.; Pauli, A.; Iberl, B.; Weigand, H.; Weis, N. Antibacterial and Antifungal Properties of Essential Oil Components. *J. Essent. Oil Res.* **1989**, *1* (3), 119–128.

(5) Mitchell, S. CLXXI.—A Method for Determining the Solubility of Sparingly Soluble Substances. *J. Chem. Soc.* **1926**, *129*, 1333–1336.

(6) Lin, H. -M; Nash, R. A. An Experimental Method for Determining the Hildebrand Solubility Parameter of Organic Nonelectrolytes. *J. Pharm. Sci.* **1993**, *82* (10), 1018–1026.

(7) Zhu, P.; Chen, Y.; Fang, J.; Wang, Z.; Xie, C.; Hou, B.; Chen, W.; Xu, F. Solubility and Solution Thermodynamics of Thymol in Six Pure Organic Solvents. *J. Chem. Thermodyn.* **2016**, *92*, 198–206.

(8) Villanueva Bermejo, D.; Angelov, I.; Vicente, G.; Stateva, R. P.; Rodriguez García-Risco, M.; Reglero, G.; Ibañez, E.; Fornari, T. Extraction of Thymol from Different Varieties of Thyme Plants Using Green Solvents. *J. Sci. Food Agric.* **2015**, *95* (14), 2901–2907.

(9) Abraham, M. H.; Kumarsingh, R.; Cometto-Muñiz, J. E.; Cain, W. S.; Rosés, M.; Bosch, E.; Díaz, M. L. The Determination of Solvation Descriptors for Terpenes, and the Prediction of Nasal Pungency Thresholds. *J. Chem. Soc. Perkin Trans. 2* **1998**, No. 11, 2405–2411.

(10) Karunasekara, T.; Poole, C. F. Determination of Descriptors for Fragrance Compounds by Gas Chromatography and Liquid-Liquid Partition. *J. Chromatogr. A* **2012**, *1235*, 159–165.

(11) Poole, C. F. Wayne State University Experimental Descriptor Database for Use with the Solvation Parameter Model. *J. Chromatogr. A* **2020**, *1617*, 1–15.

(12) Abraham, M. H.; Acree, W. E. Solute Descriptors for Phenoxide Anions and Their Use to Establish Correlations of Rates of Reaction of Anions with Iodomethane. *J. Org. Chem.* **2010**, *75* (9), 3021–3026.

(13) Manic, M. S.; Villanueva, D.; Fornari, T.; Queimada, A. J.; Macedo, E. A.; Najdanovic-Visak, V. Solubility of High-Value Compounds in Ethyl Lactate: Measurements and Modeling. *J. Chem. Thermodyn.* **2012**, *48*, 93–100.
